# Heating of an Aortic Stent for Coarctation Treatment During Magnetic Particle Imaging and Magnetic Resonance Imaging—A Comparative In Vitro Study

**DOI:** 10.1007/s00270-021-02795-4

**Published:** 2021-03-15

**Authors:** Ulrike Grzyska, Thomas Friedrich, Malte M. Sieren, Erik Stahlberg, Thekla H. Oechtering, Mandy Ahlborg, Thorsten M. Buzug, Alex Frydrychowicz, Joerg Barkhausen, Julian Haegele, Franz Wegner

**Affiliations:** 1 Department of Radiology and Nuclear Medicine, University Hospital Schleswig-Holstein, Campus Lübeck, Ratzeburger Allee 160, 23538 Lübeck, Germany; 2 Institute of Medical Engineering, University of Lübeck, Lübeck, Germany; 3 Fraunhofer Research Institution for Individualized and Cell-Based Medical Engineering, Lübeck, Germany; 4 Zentrum für Radiologie und Nuklearmedizin Rheinland, Dormagen, Germany

**Keywords:** Magnetic particle imaging, Magnetic resonance imaging, Stents, Endovascular interventions, Safety, Aortic coarctation

## Abstract

**Purpose:**

To evaluate heating of a redilatable stent for the treatment of aortic coarctation in neonates and small children in the new imaging modality magnetic particle imaging and established magnetic resonance imaging.

**Materials and Methods:**

The cobalt-chromium stent (BabyStent, OSYPKA AG, Rheinfelden, Germany) has a stent design which allows for redilatation and adjustment of the diameter from 6 to 16 mm for a use in aortic coarctation. The stent loses its radial integrity while opening at predetermined breaking points at a diameter of 14 mm or 16 mm, respectively. We measured the temperature increase in the stent at different diameters during 7-min magnetic particle imaging and magnetic resonance imaging scans with fiber optic thermometers under static conditions surrounded by air. In magnetic particle imaging, stents with diameters from 6 to 16 mm were tested while in magnetic resonance imaging only stents with diameters of 6 mm and 14 mm were investigated exemplarily.

**Result:**

In magnetic particle imaging, the measured temperature differences increased up to 4.7 K with growing diameters, whereas the opened stents with discontinuous struts at 14 and 16 mm showed only minimal heating of max. 0.5 K. In contrast to magnetic particle imaging, our measurements showed no heating of the stents during magnetic resonance imaging under identical conditions.

**Conclusion:**

The BabyStent did show only slight heating in magnetic particle imaging and no detectable temperature increase in magnetic resonance imaging***.***

## Introduction

**Fig. 1:**
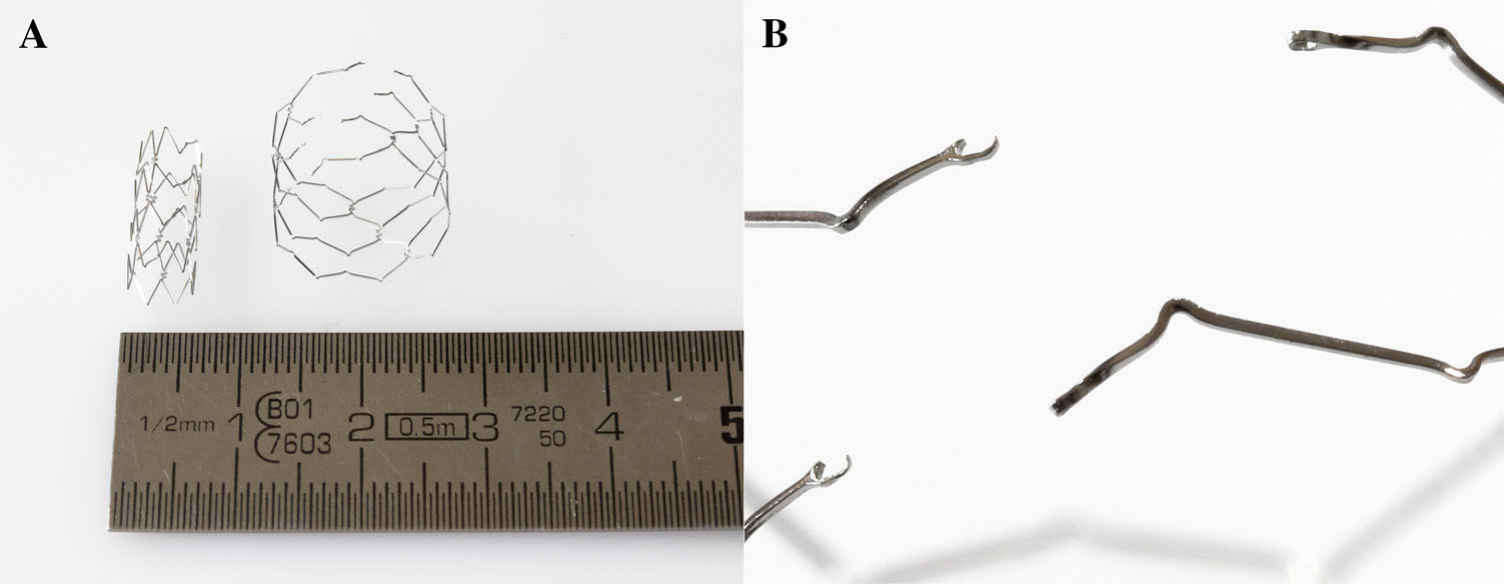
Photography of the investigated BabyStent in its native configuration at 6 mm diameter (**A**, left stent) and after stepwise dilatation to 14 mm diameter (**A**, right stent) with the struts opened at predetermined breaking points. Figure 1**B** gives a detailed view of the opened hook and eye connections

Aortic coarctation affects 5–8% of patients suffering from congenital heart disease. For a long period, surgical treatment was the only therapy [1]. There are various disadvantages of surgical treatment including immediate postoperative complications, e.g. paradoxical hypertension, injury of the recurrent laryngeal nerve or bleeding [2]. Recoarctation is the most frequent long-term complication and requires further operations [1]. Hence, in 2011, Forbes et al. reported that patients treated with endovascular stenting revealed fewer acute complications compared to surgical therapy [3]. Consequently, interventional procedures using balloon-expandable endovascular stents have gained importance in older children and adolescents [4]. In 2003, a stent design that allows for continuous dilatation after implantation and thus can be adapted to the growing vessel diameter was introduced [5]. In 2016, the principle of a breaking stent design was applied for the endovascular therapy of aortic coarctation in infants (BabyStent, OSYPKA AG, Rheinfelden, Germany) [6]. In comparison with common endovascular stents, the struts of the BabyStent carry determined breaking points. These breaking points are realized by very small hooks which can be loosened from eyelets by a balloon catheter and thus allow the stents to be stepwise dilated (Fig. 1). To our knowledge, there are no other differences in comparison to common cobalt-chromium stents. The BabyStent allows minimally invasive endovascular treatment of aortic coarctation even in young children. As this patient group is particularly sensitive to ionizing radiation, which is of course necessary during fluoroscopy-guided stent placement, magnetic resonance imaging (MRI) and magnetic particle imaging (MPI) could be very promising alternatives for guidance and visualization during aortic stenting.

MPI is a more recently introduced three-dimensional tomographic imaging modality that visualizes the spatial distribution of superparamagnetic iron oxide nanoparticles (SPIONs) by means of oscillating magnetic fields [7]. The high spatial resolution, the very high temporal resolution and the absence of ionizing radiation emphasize the potential of MPI as an imaging modality. One of the most important application scenarios is cardiovascular imaging and monitoring of endovascular interventions.

In contrast to CT and MRI, the stent lumen can be assessed without artifacts in MPI [8]. Recently, Herz et al. demonstrated the potential of MPI to guide percutaneous transluminal angioplasties of vascular stenoses [9]. Since both, MPI and MRI use oscillating magnetic fields, the heating of metallic objects is a potential risk that needs to be investigated more closely.

A systematic analysis of the heating behavior of stents with a routinely used closed stent design during MPI has indicated that the temperature increase is predominantly influenced by the stent diameter [10]. Furthermore, a non-conductive stent design with Teflon-interponates between the stent struts in longitudinal direction prevented heating despite increasing stent diameters. Hence, the breakable design of the BabyStent potentially could also influence the stents’ heating behavior in oscillating magnetic fields. Although MRI examination of patients with stents can be considered as a safe method [11], safety measurements are required for new devices.

Therefore, our in vitro study aims to evaluate heating of a redilatable stent for the treatment of aortic coarctation in neonates and small children during MPI and MRI. Naturally, the influence of the breakable stent design on heating is the main focus of this study.

## Materials and Methods

### Stents and Phantoms

**Fig. 2:**
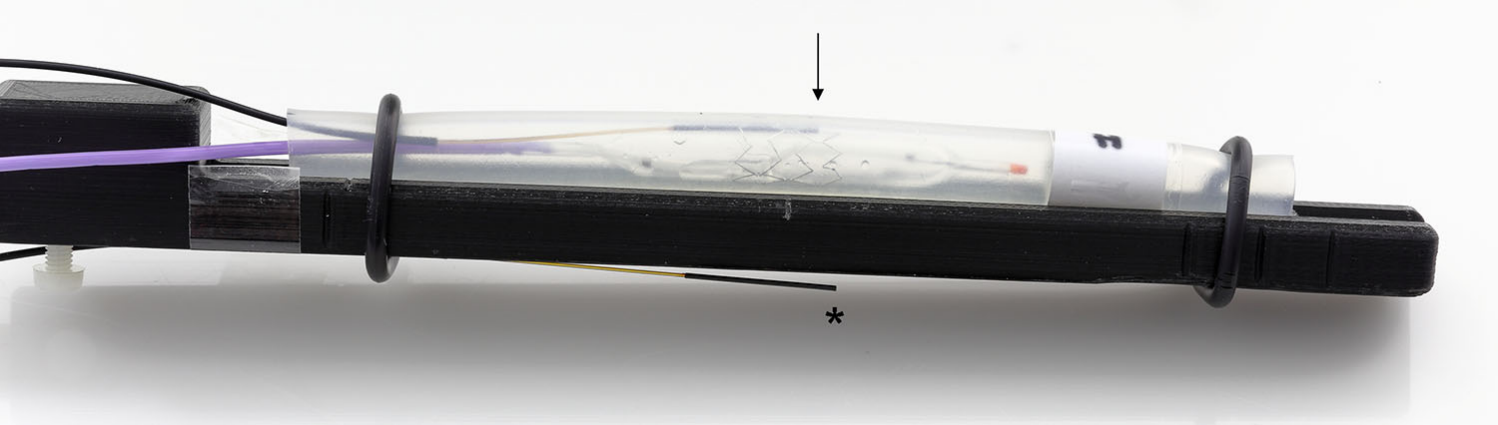
Photography of the stent in an air-filled silicone vessel phantom with a temperature probe fixed directly at the stent struts (arrow) and a reference probe at the bottom of the phantom holder (asterisk)

**Fig. 3:**
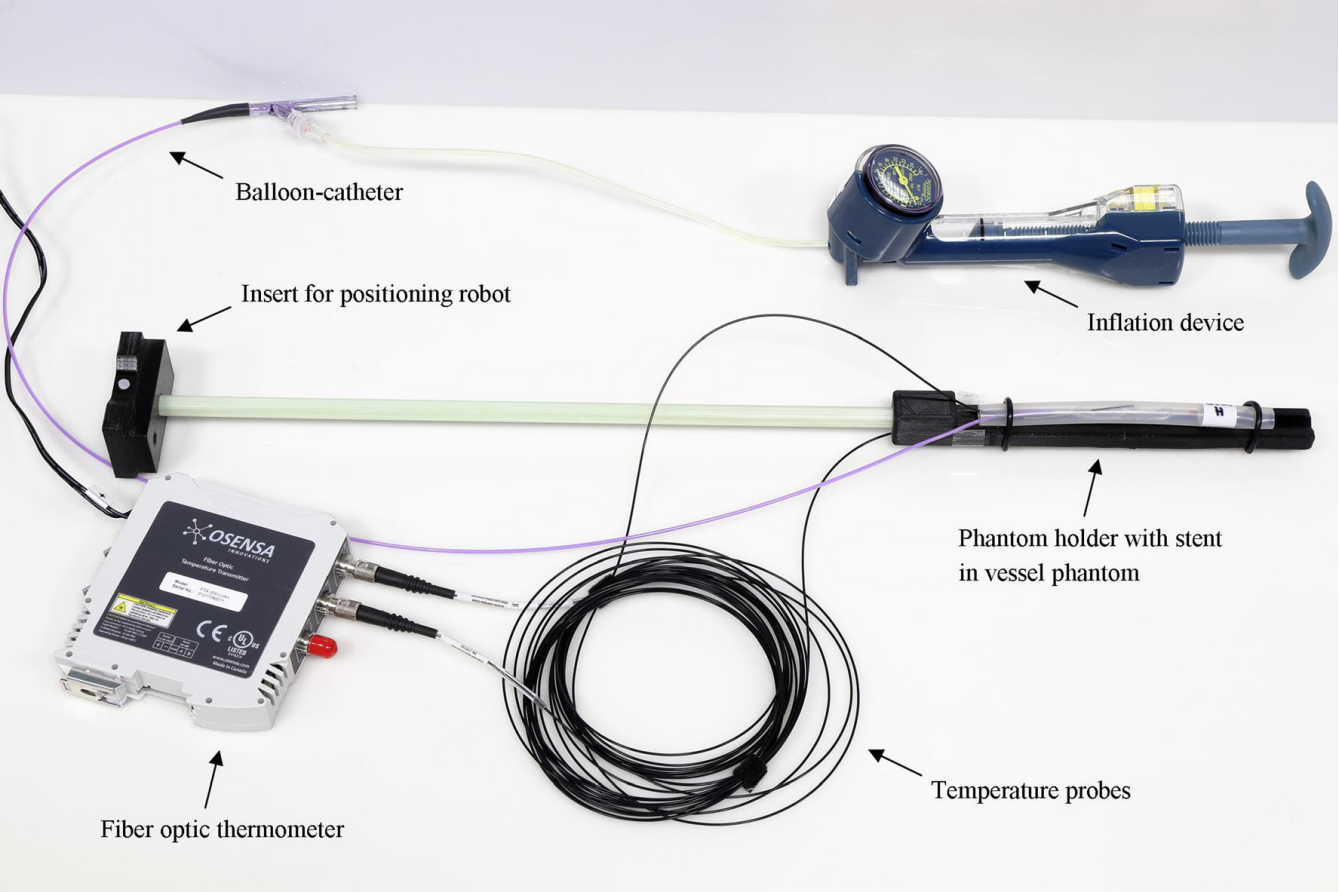
Overview of the measurement setup

**Fig. 4:**
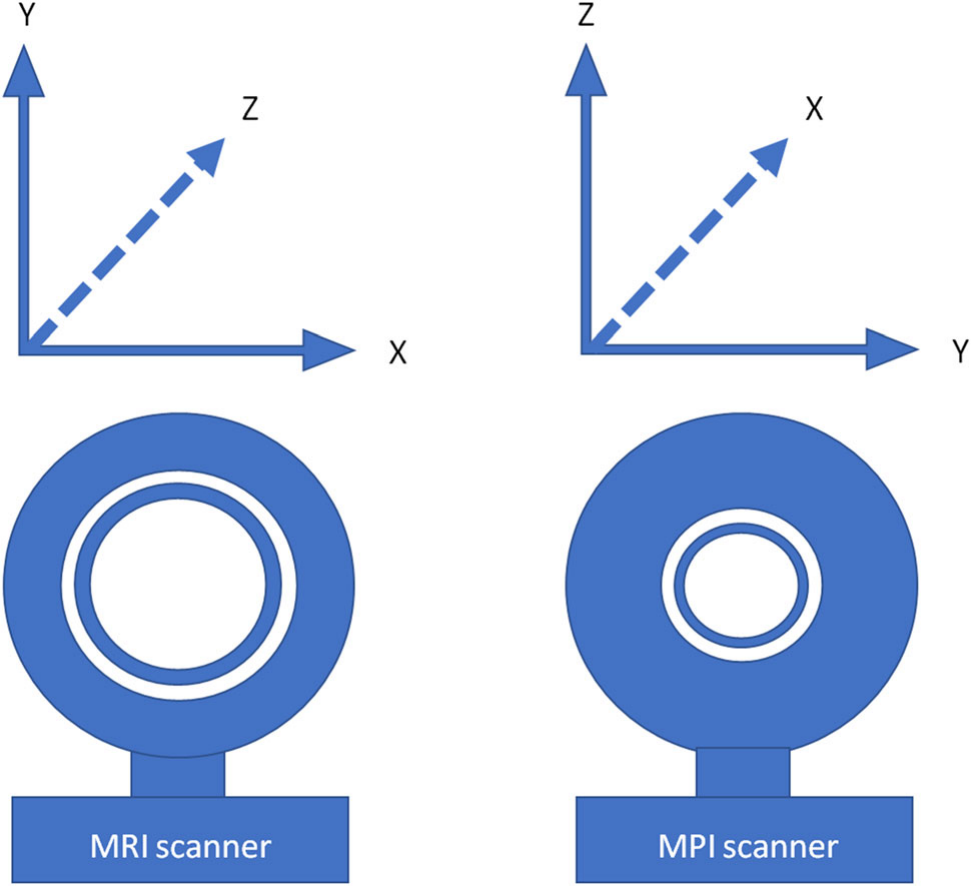
Illustration of the MRI and MPI scanners’ axes

The measurements of this work were performed with balloon-expandable cobalt-chromium stents (BabyStent, OSYPKA AG, Rheinfelden, Germany), which allow for redilatation and adjustment of the diameter from 6 to 16 mm. Reaching a certain diameter, the stent loses its radial integrity at predetermined breaking points, as shown in Fig. 1. The stent length is maximal 15 mm while the length decreases with increasing diameter. In this study, two groups of BabyStents were investigated. In the first group (Group A), the stents lost integrity at 14 mm and in the second group (Group B) at 16 mm. For each diameter of both groups, a single stent was used. To create a worst case scenario, the stents were placed in air-filled silicone vessel phantoms corresponding to the stent diameter (Fig. 2). The phantoms were aligned longitudinally to the scanner bore on a self-designed phantom holder during the measurements (Fig. 3), corresponding to alignment along the x-axis in MPI and the z-axis in MRI (Fig. 4). BabyStents of both groups (A and B) with diameters of 6, 8, 10, 12, 14 and 16 mm were tested in MPI. As there is no relevant heating of stents in MRI described in the literature, we only investigated three BabyStents with representative diameters of 6 mm (smallest stent diameter with closed struts, 6A) and 14 mm (biggest stent available with closed and opened struts, 14 A/B) in MRI as clinical reference [11, 12].

### Scanner Setup

We used a preclinical, commercial MPI scanner (MPI 25/20FF, Bruker Biospin, Ettlingen, Germany) and placed the air-filled phantoms in the center of the scanners’ field of view (FOV). The scan parameters were the following: excitation frequency: 24.5 kHz, 26.0 kHz and 25.3 kHz in *x*-, *y*-, and *z*-direction; excitation field strength: 12 mT in each direction; gradient strength: 2.5 T/m in *z*-direction and 1.25 T/m in *x*- and *y*-direction.

To correlate the heating behavior of the stents in MPI with a clinically established method, MRI measurements were performed with the identical temperature measurement setup. In MRI, the air-filled phantoms were placed in the isocenter of a clinical whole-body scanner (Ingenia 3.0 T, Philips Healthcare, Hamburg, Germany) using a 20-channel body coil. A clinically utilized T2-weighted single shot turbo spin echo sequence (TR/TE: 5073/80, FA: 90°, FOV: 220 × 220 × 42 mm, acquired matrix: 440 × 440 × 12 slices, slice thickness: 3 mm, gap 0.5 mm, TSE factor: 231) was used for imaging, because we expected a high-energy input with this sequence and therefore a potentially drastic warming. To ensure that the scanner initiates the imaging sequence, we placed two 1 l MRI phantom bottles (Phantom Bottle 1000 cc L11, Philips Healthcare, Hamburg, Germany) filled with a mixture of demineralized water, copper sulfate pentahydrate (CuSO_4_*5H_2_0, 0.77 g/l), arquad (1 ml/l, 1% solution) and sulfuric acid (H_2_SO_4_, 0.15 ml/l, 0.1 mol/l) in the scanner bore next to the vessel phantom.

### Temperature Measurements

To identify the region of the highest temperature development, pilot experiments were conducted using thermography. The stents’ middle region revealed the highest temperature increase after an exemplarily MPI scan. Consequently, for temperature measurements a fiber optic thermometer (FTX-300 lx + , Osensa, Coquitlam, Canada) was used during the MPI and MRI scans following already published measurement protocols [10, 13]. We chose a scan duration of seven minutes to represent a very long MPI imaging sequence (image acquisition time: 21 ms per frame). To guarantee comparability, we applied the MRI sequence for the same duration. The temperature probe was directly placed at the stent struts and held in place with air-filled balloon catheters. In order to measure the ambient temperature inside the scanner bore, a reference probe was attached to the bottom of the phantom holder without contact to the vessel phantom. These measured reference temperatures were subtracted from the temperatures which were measured directly at the stent struts. The maximum of the resulting temperature difference during the imaging scans was defined as (ΔT). The absolute inaccuracy of the measurement setup was 0.1 K. Consequently, we chose a temperature difference of more than 0.1 K as heating in this study.

## Results

**Fig. 5:**
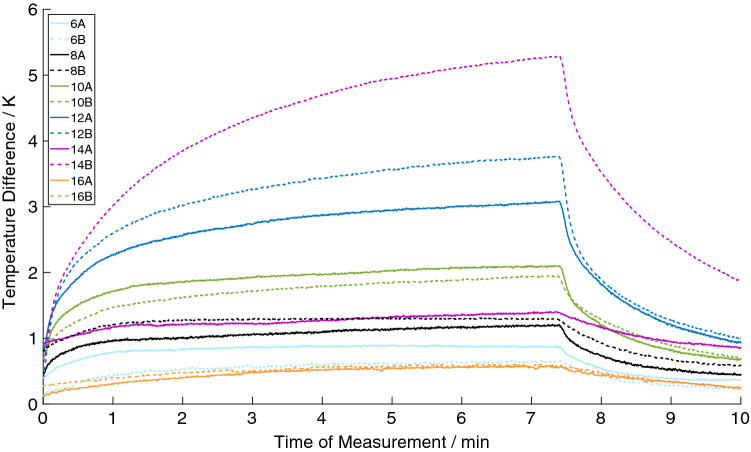
Temperature curves of the investigated stents for the MPI sequence. The nomenclature of the stents is based on the diameter (6 mm to 16 mm) and if the stent struts lost integrity at 14 mm (**A**) or 16 mm (**B**). The reference temperature was subtracted from the temperature which was measured directly at the stent struts. The up and down regulation of the drive fields takes around 20 s and has to be added to the scan duration of 7 min

In MPI, all stents showed heating (max. 4.7 K), as shown in Table 1 and Fig. 5. The temperature curves showed a monotonic increase of heating during the scans. In the first two minutes of the MPI scan, the slope of the heating curves showed the steepest increase and thus the maximum of temperature change over time, while the temperatures are approaching equilibrium after a sufficiently long experimental time. In principal, the only difference between the stents of Group A and B is the diameter at which the struts lose their integrity. Nevertheless, there is a variation of the measured temperature increase observed between identically constructed stents (e.g., stent 12 A/B: Δ*T* = 2.3 K/3.4 K).

**Table 1:** Overview of the measured temperature differences (ΔT) in MPI. The struts of the stents opened at 14 mm (Group A) or 16 mm (Group B)

Diameter (mm)	Group *A* Δ*T* (K)	Group *B* Δ*T* (K)
6	0.5	0.5
8	0.7	0.5
10	1.4	1.3
12	2.3	3.4
14	0.5	4.7
16	0.5	0.3

**Fig. 6:**
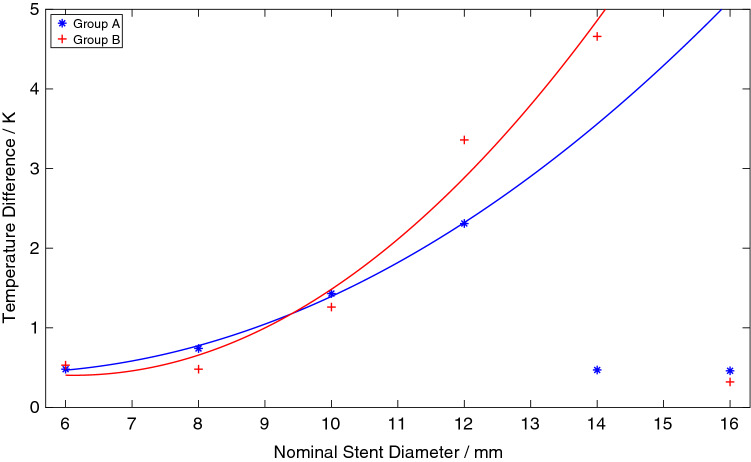
Second-order polynomial fit of the temperature difference in MPI and the nominal stent diameter showing a direct correlation between stent diameter and measured temperature difference up to the point where the integrity of the struts got interrupted. This effect can exemplarily be observed by comparing the stent 14 A and 14 B. Both have a diameter of 14 mm, but the stent with broken struts (14 A) has a lower temperature increase (ΔT = 0.5 K) compared to the stent with continuous struts (14 B; ΔT = 4.7 K)

The measured temperature differences increased in direct correlation with the stent diameter up to 12 and 14 mm, whereas the stents with discontinuous struts showed almost no heating (max. 0.5 K). Pearson’s correlation coefficient (*r*) between the diameter of the stents with closed stent struts and the measured temperature increase was *r* = 0.99 for the stents opening at 14 mm (Group *A*) and *r* = 0.96 for the stents opening at 16 mm (Group *B*) (Fig. 6).

**Fig. 7:**
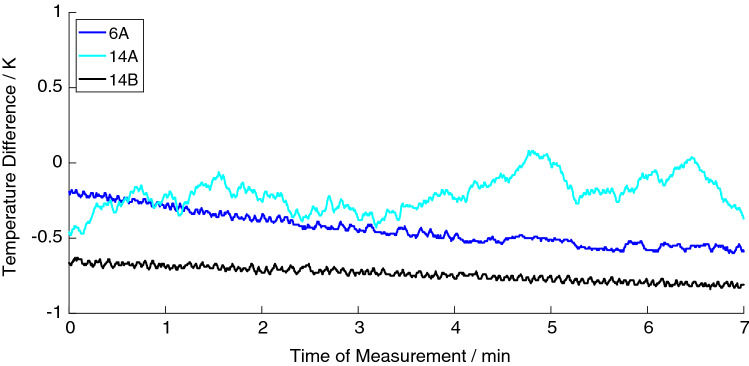
Temperature curves of exemplarily heating measurements during 7-min MRI scans. The stent with the smallest diameter and closed struts (6A) and two stents having a diameter of 14 mm, one with broken (14A) and another with closed struts (14B) were investigated in MRI

In MRI, there was no heating of the stents detected as shown in Fig. 7. The heating curves of the MRI measurements showed a slight negative slope or an undulating drift representing the cooling effect of the air conditioning system in the scanner room (Fig. 7).

## Discussion

Our study carries three messages we believe to be important. First, the BabyStent does not show any heating during MRI examinations. Second, in the MPI measurements, the heating increases up to 4.7 K with the stent diameter rising until the stent struts disconnect. In this configuration, after disconnection of the struts, the stent only shows minimal warming of 0.5 K. Third, according to our results, in terms of heating it seems safe to test the stent in future animal experiments using MPI and MRI.

The heating of ferromagnetic objects in MPI and MRI is caused by the oscillating characteristic of the applied magnetic fields. Here, two different mechanisms have to be acknowledged. First, the ferromagnetic domains of metallic objects are changing their orientations following the dynamic magnetic fields, also known as remagnetization loss. Second, since stents are made of metals, which are electrically conductive, eddy currents are induced during the imaging sequences. Following Faradays law, the amount of eddy currents increases with increasing stent diameter. This relation was proven for stents in MPI in a recently published systematic in vitro study [10]. In the MPI part of our study, this effect was also observed. In addition to the fact that a discontinuous stent design, e.g. with Teflon-interponates in longitudinal direction prevents heating [10], we show in this work that a loss of the radial integrity of the stents also causes this effect. Here, we expect that the opening of the stent struts reduces the conductivity of the stents and thus the amount of eddy currents [12]. This also reveals the dominance of the eddy current heating in MPI for stents so far.

In MRI, the radiofrequency fields in the range of MHz and the gradient pulses seem to have lower impact on the heating of ferromagnetic structures compared to MPI with oscillation frequencies around 25 kHz, because the amplitudes in MRI are much smaller. Furthermore, especially the orientation changes of the ferromagnetic domains could be suppressed by the static B0 field in MRI, magnetizing the material to saturation [10]. The literature describes only small temperature increases in endovascular stents during MRI scans [11]. Therefore, the heating behavior of stents is not a limiting factor for the safety of MRI examinations.

In our study, we did not observe any heating of the stents during the MRI scans. In MPI, the measured heating was less than 5 K. Although effects on cell replication have been described at lower temperature increases of 3.5–5 K, there are significantly fewer macromolecular changes compared to a temperature increase of > 6 K [14]. *Orihara *et al*.* also showed that a temperature increase of 6 K inhibits the proliferation of vascular smooth muscle [15]. However, because our study results present a worst-case scenario, in vivo the cooling effect of the blood flow has to be taken into account. Consequently, we assume the observed heating of stents (max. 4.7 K) in this work not to be clinically relevant. Furthermore, even in complex interventional scenarios the scanning time of a single imaging sequence should not exceed 7 min. We therefore assume the chosen scan duration in this study to be representative for a potential clinical application in both, MPI and MRI. The fact that the loss of radial or longitudinal stent integrity prevents heating could be an interesting approach for manufacturing future MPI- and MRI-compatible stent designs especially for stents with larger diameters, where the heating is more pronounced.

The guidance of vascular interventions in MRI is not fully established due to certain challenges: The image acquisition time requires the patients’ compliance or sedation of infants, respectively. Furthermore, conventional MRI scanner designs do not allow comfortable access to the patient during the intervention. In addition, all instruments and medical devices have to be MRI-compatible which strictly limits the use of established materials.

In contrast to MRI, MPI is a young imaging modality that currently holds a preclinical status. Nevertheless, it shows some characteristics that are advantageous for the use in thoracic stent implantation. The image acquisition time of a 3D volume is in the range of milliseconds and thus would not require breath hold. Furthermore, MPI allows for real-time imaging and exact stenosis quantification [16, 17], which is a prerequisite for stent implantation in patients with aortic coarctation. To guarantee free access to the patient, first concepts of single-sided MPI scanners were introduced [18].

On the way into the clinic, the enormous need of energy and the suppression of noise in the MPI signal while scaling up MPI scanners is very challenging. Most recently, a human-sized MPI scanner for brain applications was introduced [19]*.* This scanner features a field of view which would be big enough for the visualization of the human heart.

A major limitation of our study is its preclinical characteristic. In case of changing MPI parameters during the scaling up process of MPI scanners to clinical dimensions for the use with human patients, our results should be reevaluated. First MPI systems with clinical dimensions however do not exceed the magnetic field strength of the preclinical system in this study [19]*.*

To guarantee comparability of the MPI and MRI measurements, we used the same measurement setup for both modalities. By excluding any cooling effects by surrounding water or flow, we created a worst case scenario with static conditions and stents only surrounded by air, enabling the evaluation of the heating of just the stents during the imaging sequences. Thermally conductive surrounding tissue would cause a lower temperature increase, as would the presence of blood flow. Consequently, the heating of the stents on the surrounding tissue was not addressed in our study and thus should be evaluated in further studies. Nevertheless, the results need to be proven ex vivo in a human-like phantom under flow conditions as well as in vivo*,* respectively, in future studies. A direct comparison of the BabyStent with the heating behavior of other cobalt-chromium stents was not performed in this work and thus should be part of future analyses. Regarding in-stent stenting, which is necessary in full-grown adolescents who are carrying the BabyStent, the interaction of the opened stent with a closed device that can be implanted later, should be investigated concerning heating. Furthermore, a variation of MRI parameters/different sequences and their influence on the stents’ heating should be tested in further studies.

## Conclusion

The tested BabyStent showed only a presumably clinically irrelevant heating during the MPI measurements and no detectable temperature increase in MRI. Consequently, it seems reasonable to evaluate the BabyStent under more realistic conditions such as in animal experiments in MPI and MRI.
